# Serum progesterone concentration in frozen embryo transfer preparation: how (and if) we should measure it

**DOI:** 10.1016/j.xfre.2024.08.004

**Published:** 2024-08-14

**Authors:** Allison A. Eubanks, Dominque de Ziegler, Kate Devine

**Affiliations:** a National Institute of Child Health and Human Development (NICHD), Walter Reed National Military Medical Center, Bethesda, Maryland; b Fertility Clinic of Cambodia, Phnom Penh, Kingdom of Cambodia; c Shady Grove Fertility, Washington, D.C

In this issue of *F & S Reports*, Mandelbaum and Stanczyk (1) provide an excellent “minireview” of serum progesterone measurement in frozen embryo transfer cycles, exploring the nuances of assays, metabolites, timing, and actionability of measurements. They review lessons learned historically from the measurements of other sex steroids and peptide hormones and advocate for liquid chromatography–tandem mass spectrometry as the gold standard for serum progesterone measurement, despite its higher cost and technical drawbacks. The investigators appropriately highlight the lack of consensus on the optimal route and dosage of progesterone replacement, with large variability in clinical practice (1).

Recent studies have explored the role for measuring—and acting upon—serum progesterone concentration to optimize outcomes from “programmed,” also known as hormone-replaced, frozen embryo transfer cycles. European investigators have made a case for measuring serum progesterone concentration after initiation of progesterone replacement and for supplementing with subcutaneous progesterone, if the serum progesterone concentration is noted to be “low.” Importantly, these data were derived from programmed frozen embryo transfer cycles where progesterone was initiated via the vaginal route only (2).

A recent meta-analysis concluded that a serum progesterone concentration of ≥10 ng/mL was associated with optimal outcomes after programmed frozen embryo transfer. The investigators acknowledged that the data available for analysis were from studies that exhibited significant heterogeneity in timing of exogenous progesterone initiation, timing of serum progesterone measurement, route of progesterone administration, and characteristics of transferred embryos (number, stage, mode of cryopreservation, and preimplantation genetic testing for aneuploidy status). Most included studies that used only vaginal progesterone replacement. The investigators noted that “There were an insufficient number of studies administering intramuscular (IM), oral, rectal, or subcutaneous progesterone for additional analyses according to the administration route.” Notably, only 4 of the 21 included studies evaluated transfers where IM progesterone was used both before and after frozen embryo transfer for all study subjects. Two of these found higher serum progesterone concentration to be associated with worse outcomes, 1 found that the bottom quartile of progesterone concentrations experienced the worst outcomes, and 1 found no association between progesterone concentration and clinical outcomes (3). Altogether, the studies included in this meta-analysis were inconclusive as to whether the serum progesterone concentrations were predictive of outcomes in programmed frozen embryo transfer cycles where IM progesterone replacement was used.

Randomized controlled trial data from 1,060 patients undergoing frozen embryo transfer at our center showed that hormone-replaced frozen embryo transfer protocols containing IM progesterone (50 mg IM daily or 50 mg IM every third day in combination with 200 mg of micronized vaginal progesterone twice daily) provided markedly superior live birth outcomes to those using vaginal progesterone only (200 mg of micronized progesterone administered twice daily), with 5 patients being needing to receive IM progesterone to obtain 1 additional live birth (number needed to treat = 5) (4). In this randomized controlled trial cohort, the chances of having a serum progesterone concentration of <9 ng/mL (the threshold used by Labarta et al. (2)) were only 3.0% (12/399 subjects) with daily IM progesterone in oil and only 9.5% (37/388 subjects) with the combined protocol. Among these few randomized controlled trial subjects who received IM progesterone (alone or in combination) and had “low” serum progesterone concentration, 43% (21/49) of transfers resulted in live birth—not different from that of the overall cohort of subjects receiving IM progesterone or from those who received IM progesterone and had a serum progesterone concentration of ≥9 ng/mL. Here again, data did not suggest that the serum progesterone concentration predicts outcomes in programmed frozen embryo transfer protocols using IM progesterone.

Available evidence *proves* that serum progesterone monitoring is essential for determining whether to proceed with fresh embryo transfer (5). Data *suggest* benefit to measuring, and potentially acting upon, the serum progesterone concentration in the setting of programmed frozen embryo transfer cycles where only vaginal progesterone replacement is used (2). However, more research is needed to understand whether the serum progesterone concentration should be measured or acted upon when IM progesterone is administered in preparation for programmed frozen embryo transfer, as we believe to be the appropriate standard of care.

It has long been understood that vaginal progesterone administration yields higher endometrial, but lower serum, progesterone concentration than IM administration. This local transport phenomenon—dubbed “first uterine pass effect”—reassured some that vaginal progesterone replacement was sufficient. After all, should not endometrial concentrations be most relevant for determining endometrial receptivity? Hence, it came as a surprise when evidence showed that vaginal progesterone led to suboptimal results relative to IM, when used in programmed frozen embryo transfer (4). The surprise was even greater when the importance of serum progesterone concentration after vaginal administration was suggested by European investigators (2, 3). Why would serum progesterone matter if uterine concentration is high? Had we wrongly ignored that progesterone, along with its circulating metabolites, may exert systemic effects, such as triggering immunotolerance—effects diminished in the setting of vaginal administration and the “first uterine pass”?

We commend Mandelbaum and Stanczyk for their in-depth, clinically relevant review of the biology of exogenous progesterone action, metabolism, and measurement, in the context of frozen embryo transfer. They appropriately highlight the importance of progesterone assay standardization and quality control, within individual centers, from clinic to clinic, and from laboratory to laboratory. Furthermore, we call upon our field to dig deeper into frozen embryo transfer outcomes as they are correlated with the serum progesterone concentration for each type of endometrial preparation. Investigations are needed to determine when and in which protocols serum progesterone measurement helps patients. Although more research is needed, current evidence does not suffice to recommend monitoring this all-important hormone postinitiation of IM progesterone in programmed cycles.

## CRediT Authorship Contribution Statement

**Allison A. Eubanks:** Writing – original draft; **Dominque de Ziegler:** Writing – review & editing; Conceptualization; **Kate Devine:** Writing – review & editing; Conceptualization.

## Declaration of Interests

A.A.E. has nothing to disclose. D.d.Z. has nothing to disclose. K.D. has nothing to disclose.

## References

[1] Mandelbaum R, Stanczyk FZ. Progesterone in frozen embryo transfer cycles: assays, circulating concentrations, metabolites, and molecular action. F S Rep. In press. 10.1016/j.xfre.2024.05.007 PMC11456646 39381665

[2] Labarta E., Mariani G., Rodríguez-Varela C., Bosch E. (2022). Individualized luteal phase support normalizes live birth rate in women with low progesterone levels on the day of embryo transfer in artificial endometrial preparation cycles. Fertil Steril.

[3] Melo P., Chung Y., Pickering O., Price M.J., Fishel S., Khairy M. (2021). Serum luteal phase progesterone in women undergoing frozen embryo transfer in assisted conception: a systematic review and meta-analysis. Fertil Steril.

[4] Devine K., Richter K.S., Jahandideh S., Widra E.A., McKeeby J.L. (2021). Intramuscular progesterone optimizes live birth from programmed frozen embryo transfer: a randomized clinical trial. Fertil Steril.

[5] Hill M.J., Healy M.W., Richter K.S., Parikh T., Devine K., Decherney A.H. (2018). Defining thresholds for abnormal premature progesterone levels during ovarian stimulation for assisted reproduction technologies. Fertil Steril.

